# Three dimensional modeling of biologically relevant fluid shear stress in human renal tubule cells mimics in vivo transcriptional profiles

**DOI:** 10.1038/s41598-021-93570-5

**Published:** 2021-07-07

**Authors:** Emily J. Ross, Emily R. Gordon, Hanna Sothers, Roshan Darji, Oakley Baron, Dustin Haithcock, Balabhaskar Prabhakarpandian, Kapil Pant, Richard M. Myers, Sara J. Cooper, Nancy J. Cox

**Affiliations:** 1 Department of Chemical and Physical Biology, Vanderbilt University, Nashville, TN 37235 USA; 2 HudsonAlpha Institute for Biotechnology, Huntsville, AL 35806 USA; 3 Department of Biological Sciences, The University of Alabama in Huntsville, Huntsville, AL 35899 USA; 4 Department of Biostatistics, Rollins School of Public Health, Emory University, Atlanta, GA 30329 USA; 5 Biomedical and Life Sciences Division, CFD Research, Huntsville, AL 35806 USA; 6 Division of Genetic Medicine, Department of Medicine, Vanderbilt University Medical Center, Nashville, TN USA; 7 Vanderbilt Genetics Institute, Nashville, TN USA

**Keywords:** Biological models, Lab-on-a-chip, Kidney, Genetics, Gene expression, Genome

## Abstract

The kidney proximal tubule is the primary site for solute reabsorption, secretion and where kidney diseases can originate, including drug-induced toxicity. Two-dimensional cell culture systems of the human proximal tubule cells (hPTCs) are often used to study these processes. However, these systems fail to model the interplay between filtrate flow, fluid shear stress (FSS), and functionality essential for understanding renal diseases and drug toxicity. The impact of FSS exposure on gene expression and effects of FSS at differing rates on gene expression in hPTCs has not been thoroughly investigated. Here, we performed RNA-sequencing of human RPTEC/TERT1 cells in a microfluidic chip-based 3D model to determine transcriptomic changes. We measured transcriptional changes following treatment of cells in this device at three different fluidic shear stress. We observed that FSS changes the expression of PTC-specific genes and impacted genes previously associated with renal diseases in genome-wide association studies (GWAS). At a physiological FSS level, we observed cell morphology, enhanced polarization, presence of cilia, and transport functions using albumin reabsorption via endocytosis and efflux transport. Here, we present a dynamic view of hPTCs response to FSS with increasing fluidic shear stress conditions and provide insight into hPTCs cellular function under biologically relevant conditions.

## Introduction

The kidney is essential in the homeostasis regulation of the human body. It enables physiological and regulatory functions, including blood pressure regulation by controlling extracellular fluid volume, maintaining pH balance, keeping appropriate electrolyte balance, hormone production, and waste and xenobiotic removal^1^. Renal proximal tubules (PTs) of the nephron contain proximal tubule cells (PTCs), a specialized cell type responsible for active protein trafficking, and the reabsorption function of the kidney^2^. Given their essential role in drug metabolism, modeling of PTCs is particularly important for pharmacology research, as new drugs need to be tested for their effects on kidneys, especially proximal tubules, due to their increased contact with the excretion pathways involving a complex interplay of solute carrier (SLC) transporters^1,3–5^. The transporters responsible for urinary excretion of xenobiotics and the focus of pharmaceutical drug studies are the four major types of ABC efflux transporters: P-glycoprotein (P-gp, MRP1, ABCB1), multidrug resistance proteins 2 and 4 (MRP2 and MRP4, ABCC2/4), and breast cancer resistance protein (BCRP/ABCG2)^4–6^. Together, these efflux transporters eliminate a variety of xenobiotics^7,8^. Besides waste product excretion and reabsorption of filtered solutes by transporter pathways, PTCs use endocytosis to recycle nutrients and proteins from the filtrate, such as glucose and albumin. The proximal tubule function is both complex and crucial for normal kidney function.

Simple monolayer culture systems are widely utilized for the understanding of kidney functions, including drug binding and toxicity. However, alternative models that more closely replicate the in vivo environment are likely to further our ability to recapitulate the in vivo functions of reabsorption and transportation and better understand how they breakdown in disease. Previous studies of cell culture models show that PTCs studied in traditional, static cell culture often lack or rapidly lose critical phenotypic and functional aspects, such as cell polarity and receptor-mediated transport^9,10^. However, 3D models for PTCs mimic the in vivo environment by exposure to Fluid Shear Stress (FSS). These models mimic the glomerular filtrate flow, which is present within the lumen of the PTs as the ultra-filtrate passes through to eventually reach the urinary bladder to be excreted^9,11^. Cells cultured under continuous FSS and three-dimensional (3D) flow models have gained increasing interest due to their ability to recreate precise cellular organizations and previous work has shown that PTC cultures exposed to FSS in a 3D model more closely recapitulate in vivo PTs morphology and function; however, these studies have explored widely differing FSS used on diverse cell types (varying between 0.01 dyn/cm^2^ to 5.6 dyn/cm^2^)^12–15^. In humans, a healthy FSS is estimated to vary between 0.3 dyn/cm^2^ and 1.2 dyn/cm^2^, but can reach high levels of about 1.6 dyn/cm^2^ and lower levels (below 0.5 dyn/cm^2^) in individuals with renal disease^1,16^. PTCs under fluidic shear have increased transporters’ functionality and demonstrate changes in expression of key genes such as solute carrier (SLC) and ABC efflux transporters^3,17^. Nevertheless, the global impact of FSS exposure on human gene expression remains largely unknown, and the effects of FSS at differing rates on the transcriptional profiles in human kidney proximal tubule cells has not been investigated. Furthermore, previous studies of PTCs have used PTs from model and non-human organisms; they showed that the cells were lacking important transporters, receptors, or other physiological attributes^2^. To overcome some of these shortcomings, we studied a novel, reproducible 3D model of human cells derived from proximal tubules that closely the mimics function and transcriptional profiles of primary proximal tubule cells. Our model system uses an immortalized human PTC line (RPTEC/TERT1) combined with a highly reproducible, microfluidic platform developed by our group^6,18^.

We characterized epithelial monolayer of RPTEC/TERT1 cells exposed to varying levels of fluid shear stress in a 3D model by using RNA-sequencing and microscopy to demonstrate that this model is excellent for understanding PTC function. We explored transcriptional changes following growth under physiological fluid shear stress of 0.1 dyn/cm^2^, 0.25 dyn/cm^2^, and 0.5 dyn/cm^2^. At this physiologically-relevant level of fluid shear stress, we assessed cell morphology, presence of cilia, and transport functions such as endocytosis and efflux transport. This detailed characterization demonstrates that our 3D model provides a platform for studying human kidney biology and global genomic factors contributing to PTCs function, indicating that it can serve as a useful tool for evaluating renal biology, pathophysiology, and pharmaceutically-induced nephrotoxicity.

## Results

### Fluid shear stress affects transcriptomic profiles in human kidney proximal tubule cells

**Figure 1:**
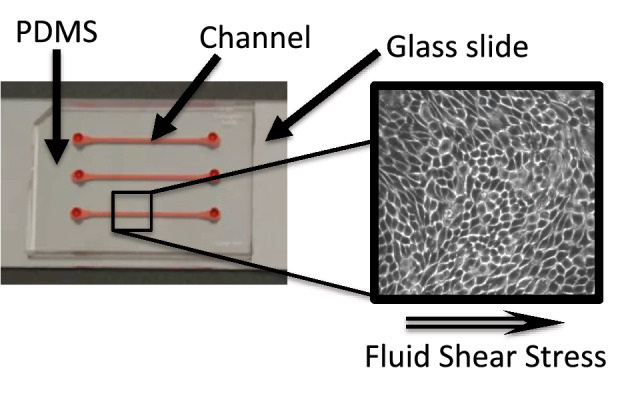
Aerial perspective image of linear kidney proximal tubule device. The linear device is assembled on a microscope glass slide with polydimethylsiloxane (PDMS) creating three of the channel walls (red) allowing three independent channels for cellular experiments. Dimensions of cell culture channels (highlighted in red) are 500 µm × 100 µm × 1 cm (width × height × length).

To study global gene expression changes induced by fluid shear stress (FSS), we used RNA-sequencing to measure transcriptomic profiles of human proximal tubule-like epithelial cells (RPTEC/TERT1 cells) after 24 h of exposure to fluidic shear stress of 0.1 dyn/cm^2^, 0.25 dyn/cm^2^, or 0.5 dyn/cm^2^ using a 3D microfluidic chip (Fig. 1). The chip is composed of three parallel channels that allow three-dimensional growth in a tubule-like shape. We compared each of these FSS conditions to control channels on the same chip maintained under static conditions. We isolated total RNA from these cells and performed RNA-sequencing. We analyzed the FSS treated samples compared with the static controls to elucidate the overall transcriptomic profiles altered by FSS. We identified 10,444 genes that are significantly differentially expressed (p-adjusted < 0.05) between the 12 FSS samples and 10 static control samples (Supplementary Dataset File S2). Of these significantly differentially expressed gene, approximately 56% were up-regulated (5605) and 46% were down-regulated (4839). The top 15 most significantly expressed genes that are involved in the regulation of proximal kidney tubule function are listed in Table 1. Hierarchical clustering representing gene expression level of all 22 samples showed two distinct clusters (log2FC >  ± 1; p-adjusted < 0.05): one with FSS-treated samples and the other one with static controls (Supplemental Figure S1).

**Table 1:** Top significantly expressed genes involved in regulation of proximal tubule regulation.

Gene Symbol	padj	log2FC	Relationship to proximal tubule cells
AKR1C1	1.60E-142	2.961	Regulation of aldo–keto reductases
CNNM4	9.83E-131	1.167	Mg2R homeostasis
AKR1B10	1.88E-112	4.319	Mitochondrial aldo–keto reductases with activity towards steroids and 3-keto-acyl-CoA conjugates
SLC43A2	3.15E-109	1.937	Essential amino acid transporter (Lat4)
CYP4F11	3.96E-109	2.659	Cytochrome P450 (CYP) enzymes
CYP4F3	6.83E-108	6.305	Cytochrome P450 (CYP) enzymes
PLAU	4.64E-105	-2.089	Preventing calcium salt precipitation
CLCN2	1.50E-91	1.175	Chloride channel 2 (ClC-2)
SLC44A2	1.32E-87	2.020	Drug transporters of the organic anion transporter (OAT) family
NGFR	1.56E-81	5.850	Rapamycin-induced autophagy protects proximal tubular renal cells against proteinuric damage
TRIM16L	3.29E-80	2.154	Regulation of response to stimulus
CABYR	9.63E-69	2.270	Expressed in testes and ciliated cells
TNFRSF10D	9.95E-65	2.278	Biomarker for tubulointerstitial injury
CACNB3	2.23E-62	-0.900	Calcium channel
TRIOBP	3.43E-62	-0.887	Regulation of SGLT expression

**Figure 2:**
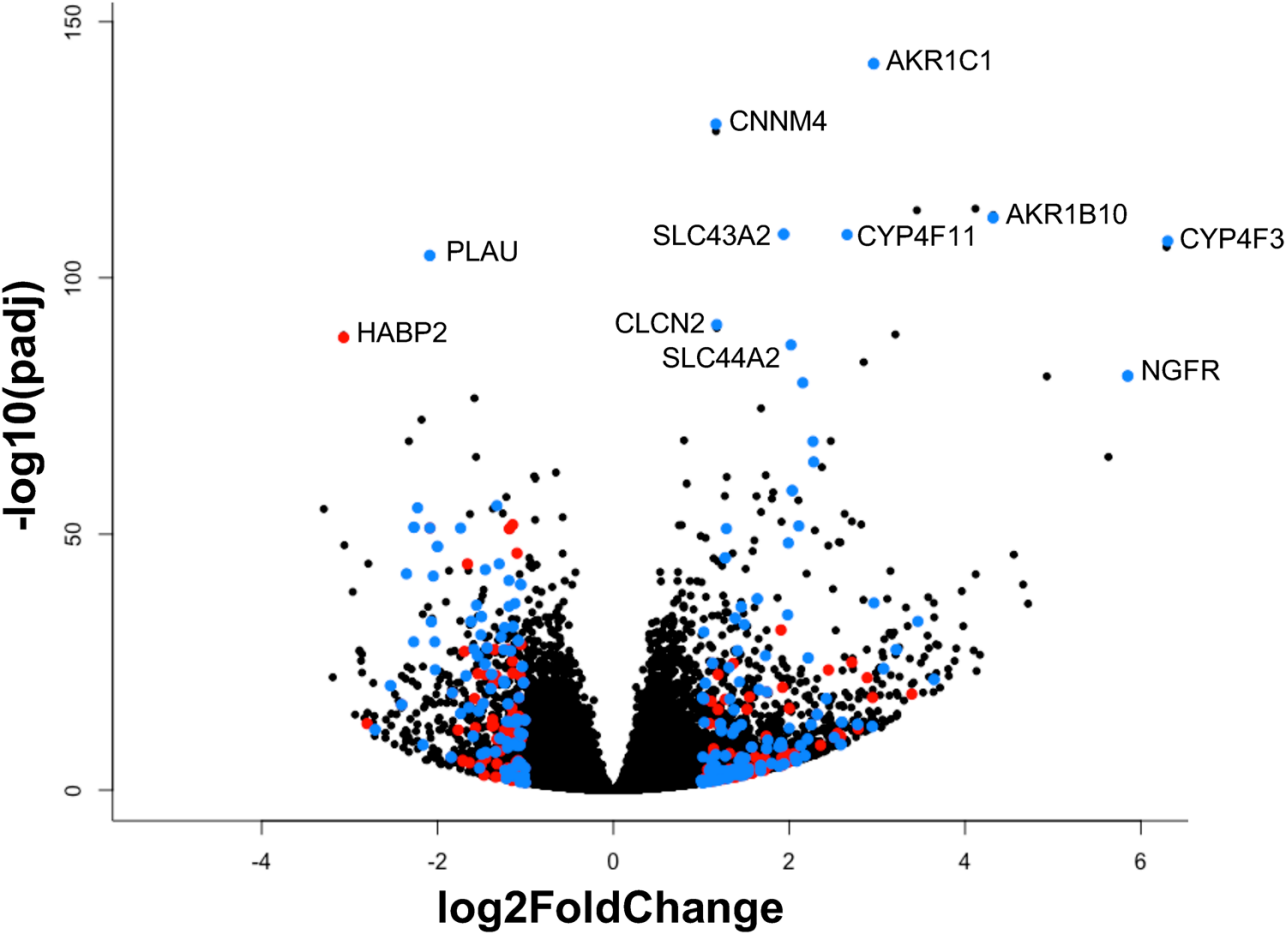
Volcano plot displaying differential expressed genes in samples under fluid shear stress conditions and static environment. The y-axis corresponds to the mean expression value of -log10 (padjust value), and the x-axis displays the log2 fold change value. Blue colored points represent most significant genes involved in kidney proximal tubule function. Red colored points represent GWAS (genome-wide association study) hits that denote inherited genetic variants associated with risk of renal diseases. Both subsets groups have padj equals < 0.05 and have a log2FC >  ± 1.

The genes we identified through transcriptomic analysis reveal both known and novel connections to human PTCs function and dysfunction. The volcano plot highlights a subset of highly differentially expressed genes associated with PT genes and previous kidney genome-wide association studies (Fig. 2). Differentially expressed genes observed upon flow treatment revealed 339 genes that have previously been cited in the literature as related to or important for PTs structure, function, and regulation of the cell system (Fig. 2, blue). Notably, these genes are involved in the regulation of aldo–keto reductases, magnesium homeostasis, PTCs transporters (including solute carrier family), ATP-binding cassette (ABC) transporters, renal drug metabolism, and cilia (Supplementary Dataset File S3). Furthermore, 156 genes were nearby GWAS (genome-wide association study) hits that denote inherited genetic variants associated with the risk of renal diseases, (Fig. 2, red). Relevant diseases and symptoms associated with one or more relevant phenotypes described in the NHLBI-GRASP GWAS catalog from the GWAS include, chronic kidney disease, filtration rate, albuminuria, proteinuria, and urinary metabolite imbalance (Supplementary Dataset File S4).

### Pathway analysis of associated genes of fluid shear stress treated cells

To obtain insights into biological processes influenced by FSS, we used LRPath to determine pathways enriched among our differentially expressed genes. Our analysis of 10,444 significantly differentially expressed genes, up- and down-regulated revealed significant enrichment of expected and novel pathways (GO Analysis Supplementary Dataset File S5-S7). As expected, pathways known to be important for kidney proximal tubule cell function, such as signal transduction, metabolism, cytokine signaling, cell–cell/matrix interaction, tight junction molecule, cell adhesion molecules, extracellular matrix components, and pathways which respond to a stimulus, such as endocytosis were enriched. Furthermore, our data show that genes differentially expressed after treatment with fluid shear stress included critical genes for PTCs function. These included CYP4F3 and CYP4F11, which encode members of the cytochrome P450 superfamily of enzymes involved in the metabolism of fatty acids, xenobiotics, therapeutic drugs, and signaling molecules, and transporters such as SLC47A1 and SLC47A2 which are important for renal excretion of diverse substrates (including drugs), a canonical feature of these cells. Importantly, these genes are not readily expressed in RPEC/TERT1 cells without the environment of fluid shear stress as observed in the Human Protein Atlas cell RNA expression data (www.proteinatlas.org). Additionally, we observed changes in cell adhesion molecules CLDN2 and CLDN16 which function in tight junction formation critical for role in epithelial barrier function. Defects in these junctions can cause a wide spectrum of kidney diseases, such as hypomagnesemia, hypercalciuria, kidney stones, and hypertension. Furthermore, genes involved in the PT bicarbonate reclamation pathway (SLC4A4 and PCK1) are affected by FSS. This pathway is used by proximal tubules to reabsorb approximately 80% of the filtered bicarbonate (HCO^3−^) as well as generating new bicarbonate for regulating blood pH.

**Figure 3:**
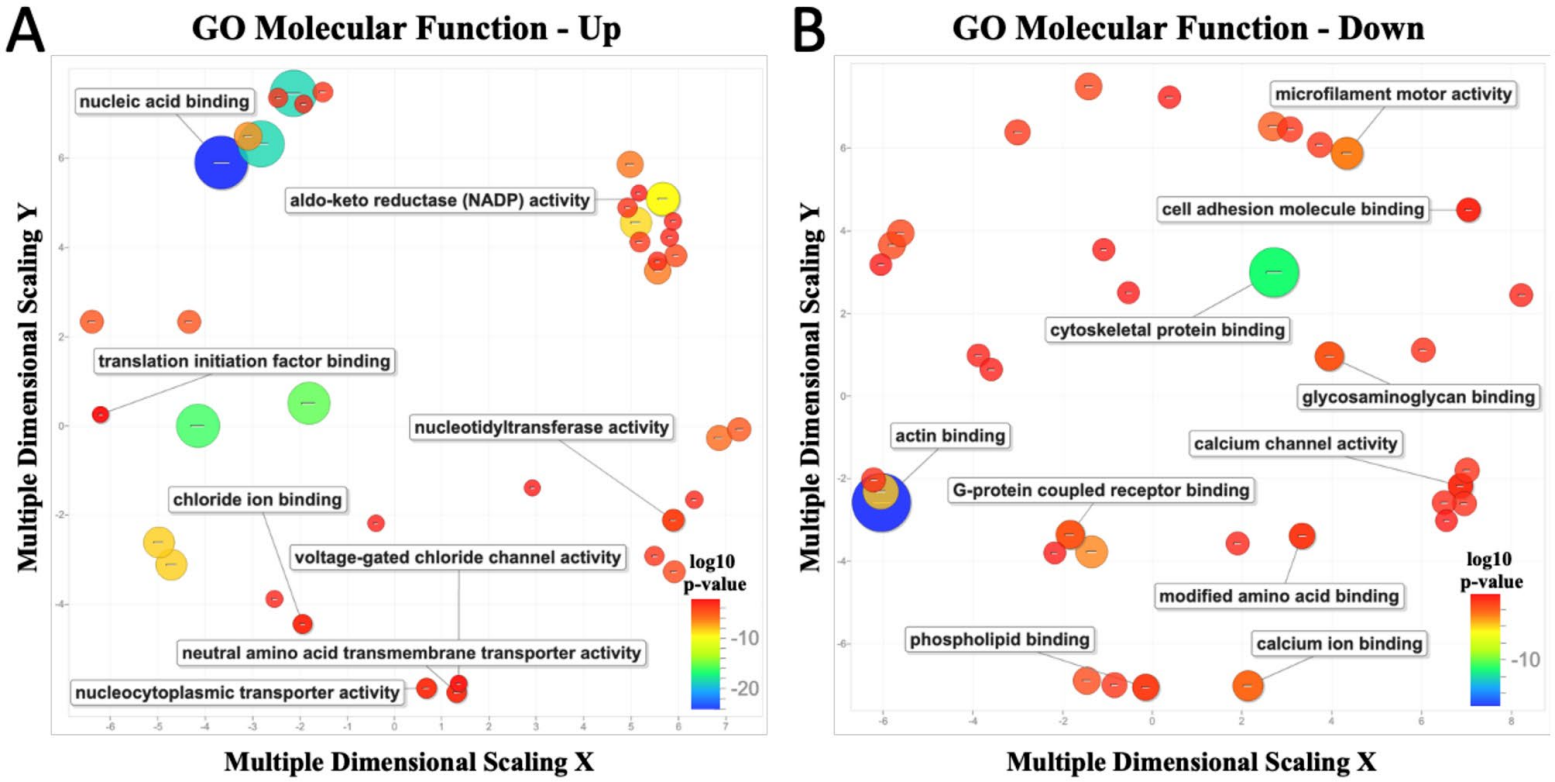
GO enrichment analyses of FSS treated and static controls. Scatterplot of enriched pathways for a subset of up-regulated genes (**A**) and down-regulated (**B**). To identify similar GO terms among the enriched terms, these were categorized using semantic clustering (REVIGO). The GO terms within the scatterplot are represented as a circle and are related to each other and to a similar process. The circle size symbolizes the amount of GO terms grouped within that cluster and colorization corresponds to the p-value of the enrichment analysis. The red color indicates the highest *p* value, and the blue color the lowest p-value.

To summarize and visualize GO terms among the enriched pathways, we used the REVIGO tool to determine semantic clustering of functional categories for up-regulated genes (Fig. 3A) and down-regulated genes (Fig. 3B)^19^. Multiple anticipated GO terms associated with PTCs were shown to be enriched and up-regulated, such as channel and transport activity, and nucleic acid binding. Notably, pathways involving cytoskeletal, actin, cell adhesion, and specific channel activity and binding pathways were generally down-regulated under FSS conditions.

### Identifying flow dependent gene expression changes

**Figure 4:**
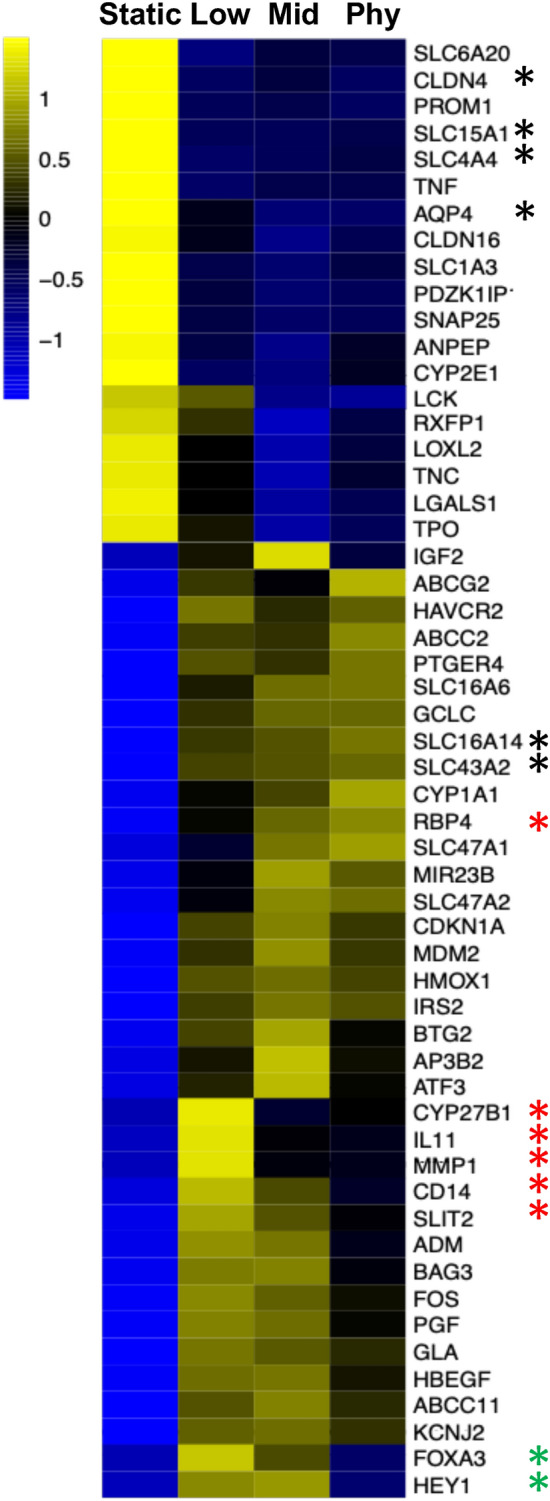
Proximal tubule cells under different fluidic shear stress alter expression of example genetic profiles. Statistically significant changes in expression of example genes associated with proximal tubule cells were identified by cross-comparisons of all four conditions (Low (0.1dyn/cm2), Mid (0.25dyn/cm2), Physiological (Phy) level (0.5dyn/cm2), and static baseline). Each column represents data from biological replicates at a specific experimental fluidic shear stress. Example genes were grouped by subtype or directionality. Replica count data for each gene were average across all corresponding samples and plotted as a heat map. More significant values are visualized in the yellow range and lower, less significant values, are visualized in blue.

We examined a set of PT gene expression patterns to identify genes whose expression changes as a function of FSS (Fig. 4). As with the previous analysis, this approach identified genes important for proximal tubule function and regulation and were affected by FSS in an overall significant gene expression change compared to static. Some examples include ones from the solute carrier (SLC) and claudin gene families, and Aquaporin-4 (AQP4) (e.g. black asterisks). However, expression levels of other genes were impacted on by the shear stress level. Some notable impact of individual FSS on expression are within groups in which the gene expression was increasing or decreasing coordinately with FSS (red asterisks, CYP27B1, IL11, MMP1, CD14, SLIT2). In some cases, gene expression was impacted in opposite directions at low and high FSS values. (FOXA3 and HEY1, green asterisks). This indicates that the specific FSS impacts gene expression.

### Proximal tubule morphology and molecular markers with the treatment of fluid shear stress

**Figure 5:**
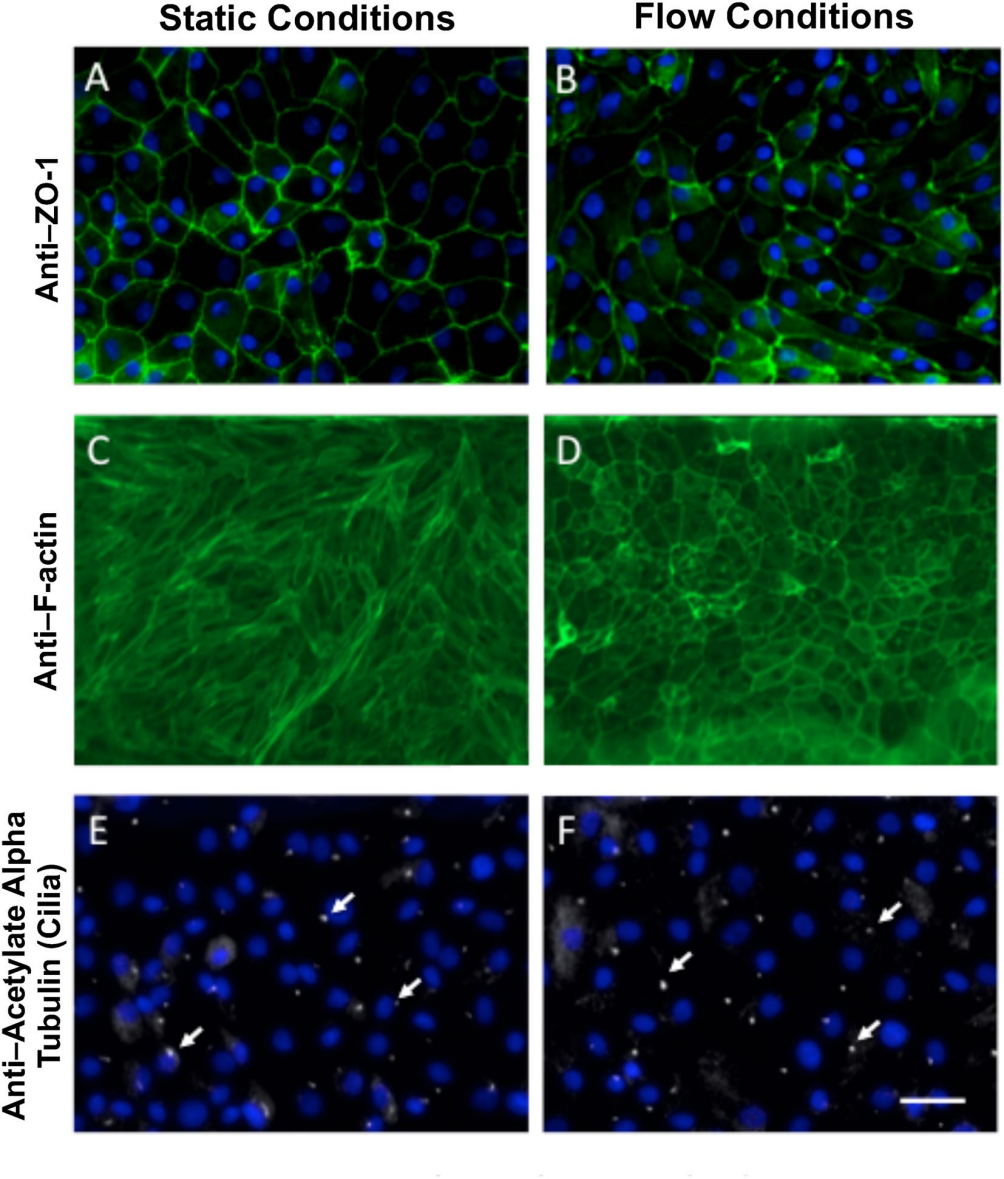
Human kidney proximal tubular epithelial cell morphology 24 h under static conditions versus fluid shear stress. Immunofluorescence staining of the tight junction protein ZO-1 (tight junctions) (green) and DAPI (blue) in static (**A**) and flow cells (**B**). Immunofluorescence images of F-actin under static conditions (**C**) and after 24 h of exposure to fluid shear stress (**D**). Under the shear stress, F-actin reorganizes at the periphery of the cells (**D**). Immunofluorescence staining of acetylated tubulin (white) to visualize primary cilia and DAPI (blue) under static (**E**) and flow (**F**) remain similar under both conditions. (Bar = 50 µm).

Immunofluorescence microscopic images of the proximal tubule cells after 24 h of FSS revealed well defined characteristic morphological properties of a confluent epithelial monolayer lined by a continuous, unaltered linear distribution of the tight junction protein, zonula occludens-1 (ZO-1), under both FSS and static culture conditions (Fig. 5A,B). The ZO-1 (green) outlines each of the cells and surrounds the DAPI labeled nuclei (blue). Consistent expression of the tight junction marker protein ZO-1 in confluent cell monolayers of PTC under both conditions displayed the integrity of intercellular junctions found within healthy, functioning cells. In contrast, treatment with physiological fluid shear stress (0.5 dyn/cm^2^) induced noticeable rearrangement in actin cytoskeletal properties (F-actin) of the cells compared to the static channel (Fig. 5C,D). Similar to observations in animal and human PTC, the FSS caused the F-actin to reorganize to the periphery of the cell compared to diffuse labeling across the cell observed under static conditions. The apical localization of F-actin and intracellular tight-junction protein (ZO-1) indicates appropriate cell polarization in this platform. Furthermore, we found that exposure to FSS left the primary cilia intact (Fig. 5E,F). This demonstrates that the primary cilia remain on the RPTEC/TERT1 cell surface, even after the 24-h treatment of FSS. These data complement the expression data showing that genes corresponding to proximal tubule morphology (tight junctions, F-actin, and cilia) were differentially expressed with FSS (p-adjusted < 0.05, |log2FC|> 1) (Table 2). These include genes from LPS-TNF-α-ERK1/2 signaling pathway (TNF) and myosin motors (MYO7B) and/or scaffolding complexes (SGK), which likely play a role in receptor-mediated endocytosis in the PTs^17^.

**Table 2:** Expression of selected genes with mechanistic and functional relevance to tight junctions, f-actin, and cilia in proximal tubules.

Gene symbol	padj	log2FC	Description
**Tight Junction and Proximal Tubule**			
CLDN16	2.35E-23	-1.384	Claudin 16
TNF	7.24E-17	-1.643	Tumor necrosis factor
ABCG2	1.17E-07	1.164	Breast cancer resistance protein (BCRP)
CLDN2	1.85E-05	-1.099	Claudin 2
**F-actin and proximal tubule**			
HPSE	1.31E-33	-2.0710	Heparanase
ABCC2	7.27E-22	1.4333	Multidrug resistance protein 2 (MDR2)
CCL2	9.78E-08	-1.4992	Collagen type I alpha 2 chain
MYO7B	1.44E-09	-2.166	Myosin VIIB
COL1A2	0.03265	1.0195	Collagen type I alpha 2 chain
**Cilia and proximal tubule**			
ITPKB	8.19E-21	-1.363	Inositol-trisphosphate 3-kinase B
CLDN2	1.85E-05	-1.099	Claudin 2

Tables of differential expressed genes corresponding to morphological terms that were highly significant (padj < 0.05).

### Fluid shear stress induces genes corresponding to the endocytosis process and reabsorption

**Figure 6:**
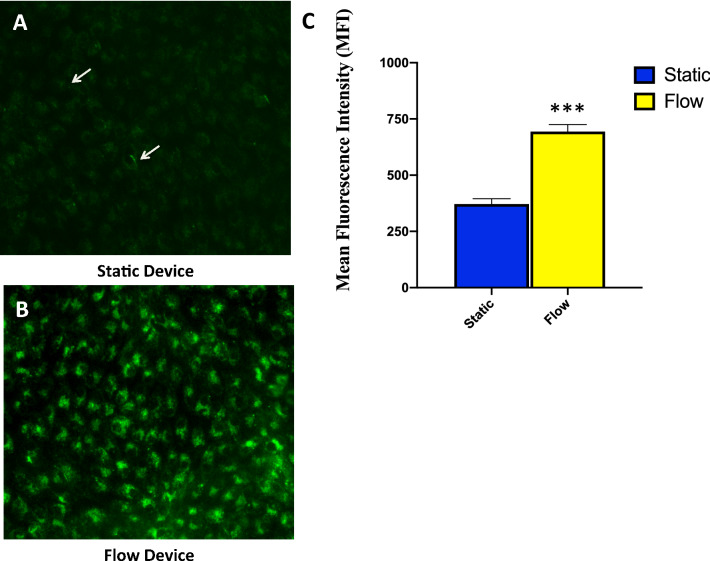
Analysis of human proximal tubular albumin reuptake function. Albumin uptake by human proximal tubular epithelial cells after 15 min incubation at 37 °C with 50 µg mL-1 of FITC-albumin (green) added to the channel under static conditions (**A**) and fluid shear stress conditions (**B**). Mean Fluorescence Intensity (MFI) of RPTEC/TERT1 cells 24 h after static conditions versus fluid shear stress conditions showing a significant increase in FITC-Albumin fluorescence signal (**C**) (***p ≤ 0.001). Transcriptome profiling provides insight to differentially expressed genes corresponding to endocytosis process.

Many large, soluble molecules are reabsorbed in the PTCs by receptor mediated endocytosis as a part of essential renal physiology. A subset of genes induced by fluid shear stress (padj < 0.05 and a log2FC >  ± 1) are important for endocytosis (Table 3). These genes are involved in endocytic vesicle coat proteins, lysosomal storage, and receptor families. We tested whether there was evidence for altered endocytic function. Reabsorption of plasma proteins from the glomerular filtrate can be modeled in vitro by monitoring FITC-conjugated albumin uptake by PTCs. We measured cellular albumin uptake of the human RPTEC/TERT1 cells in the device after 24 h of physiological FSS or static conditions. Cells were removed from the flow and treated with FITC-conjugated albumin and uptake was measured using fluorescence microscopy (Fig. 6A,B). Uptake of FITC-conjugated albumin of the cells under FSS was significantly higher compared with cells grown under static conditions (Fig. 6C , p < 0.001, Student’s *T *test). The increased transport of FITC-albumin activity we observed was likely mediated by an increased delivery due to flow dynamic mechanisms, as seen in in vivo conditions. Our data are also consistent with previous studies performed on proximal tubules, where fluid shear stress-induced mechanisms have been shown to increase function^20,21^.

**Table 3:** Expression of selected genes with relevance to the process of endocytosis in proximal tubules.

Gene Symbol	padj	log2FC	Description
SLC19A3	1.58E-12	-2.709	sodium–hydrogen exchanger 3 (NHE3, solute carrier family)
SHH	1.61E-28	-1.4367	sonic hedgehog signaling molecule
UGCG	2.67E-25	-1.4590	UDP-glucose ceramide glucosyltransferase
CYP2E1	3.13E-24	-2.0254	cytochrome P450 family 2 subfamily E member 1
CLDN16	2.35E-23	-1.3842	claudin 16
EGF	1.62E-18	1.0292	epidermal growth factor
HMOX1	1.66E-18	1.3240	heme oxygenase 1
ABCG2	1.17E-07	1.1638	breast cancer resistance protein (BCRP)
TG	1.89E-06	1.4330	thyroglobulin
CLCNKA	0.005725	-1.0687	chloride voltage-gated channel Ka
NPHS1	0.02228	1.0809	NPHS1 adhesion molecule, nephrin

Tables of differential expressed genes corresponding to morphological terms that were highly significant (padj < 0.05).

### Expression of xenobiotic efflux transporters changes with fluid shear stress

**Figure 7:**
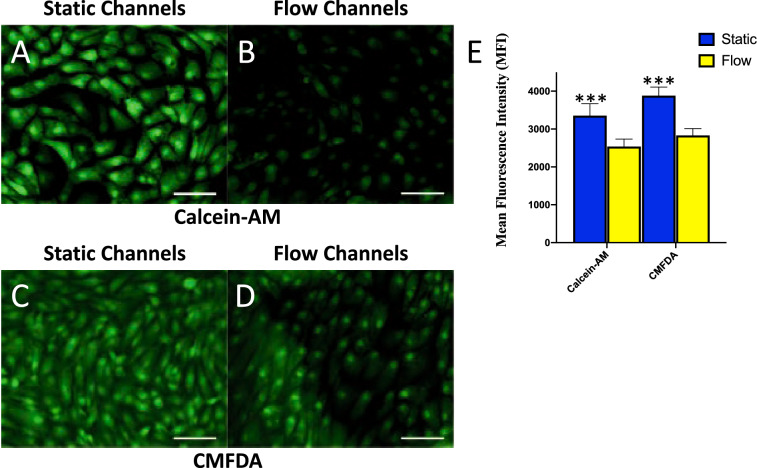
Fluorescent transporter substrate Calcein-AM and CMFDA amasses in RPTEC/TERT1 cells under static conditions and dissipates with the application of FSS. Figure (**B**) and (**D**) sample fluorescence images demonstrate decreased in Calcein-AM and CMFDA accumulation in cells after 24 h of FSS treatment prior to staining compared to static channels (**A** and **C**) (Bar = 100 µm). (**E**) Quantitative analysis of Mean Fluorescence Intensity (MFI) of Calcein-AM showed a significant decrease after cells were treated with 24 h of FSS versus static environment (yellow filled bars). Indicating the increased efflux activity after being kept under fluidic conditions (Bar = 100 µm; n = 4, ***p ≤ 0.001).

Transporters that mediate substrate efflux, such as the ATP-binding cassette transport proteins (ABC transporters), are critically important for the canonical PTCs function of xenobiotic efflux^21,22^. Our transcriptomic analysis shows that expression of genes involved in solute carrier (SLC) and ATP-binding cassette (ABC) drug transporters (including the Multi-drug Resistance transporters, MDRs) dramatically change under shear stress with the treatment of FSS (Table 4). We used fluorescence image analysis to measure changes in efflux transport activity with FSS exposure (Fig. 7). We quantified cellular accumulation of the dyes Calcein-AM, and CMFDA in cells that have been maintained for 24 h in our device/model under physiological fluid shear stress (0.5 dyn/cm^2^) to those maintained under static conditions. As transporters actively efflux the substrate, a decrease in fluorescence is a measure of increased transporter activity. The fluorescent substrate accumulation in the cells cultured under FSS decreased in both Calcein-AM (Fig. 7A) and CMFDA (Fig. 7C) compared to static controls (Fig. 7B,D, respectively). Decrease of fluorescent substrate accumulation were quantified and demonstrated graphically (Fig. 7E , *p* < 0.001, Student’s T-test). As in the case for FITC-albumin uptake rate increase into the cells, these indicate that kidney PTCs under fluidic flow stress display more effective efflux activity of P-glycoprotein (P-gp, MRP1, ABCB1) transporter function and multidrug resistance proteins 2 and 4 (MRP2 and MRP4, ABCC2/4), (using Calcein-AM and CMFDA substrates respectively). Furthermore, we found that of the 193 genes specifically associated with PTCs in Rat PT regions S1, S2, and S3, over half (90/193) are expressed at an RPKM of 0.5 (a common standard for reliably detected expression) in our RPTEC/TERT1 cells (Supplementary Dataset File S8)^23^. Due to the fact that these and other transporters are critical for identifying and understanding drug toxicities and drug–drug interactions, this human PTCs microfluidic device provides an advantageous in vitro model for renal physiology, kidney diseases research, pharmaceutical and nephrotoxicity studies.

**Table 4:** Significant expression of genes with importance transportation in proximal tubules.

Gene Symbol	padj	log2FC	Description
SLC43A2	3.1E-109	1.937	solute carrier family 43 member 2
SLC15A1	6.43E-52	-2.086	solute carrier family 15 member 1
ABCA3	7.15E-41	-1.053	ATP binding cassette subfamily A member 3
ABCC2	7.27E-22	1.433	multidrug resistance protein 2 (MDR2)
ABCB1	2.26E-09	-1.085	P-gp, multidrug resistance protein 1 (MDR1)
ABCG2	1.17E-07	1.164	breast cancer resistance protein (BCRP)

Tables of differential expressed genes corresponding to morphological terms that were highly significant (padj < 0.05).

## Discussion

Renal epithelial cells play a critical role in kidney function and undertake the majority of the kidneys’ reabsorptive and secretory burdens. Dysfunction of these cells leads to improper filtration of the proximal tubule and ultimately results in kidney disease. A model of the proximal tubules' normal cellular function under physiological fluid shear stress can add value to in vitro drug safety assessments and pathobiology research. While other in vitro models have been able to reproduce kidney and PT environments, there are limitations by variable channel size, FSS applied to classical 2D system hybrids and device designs that are difficult to reproduce. Here we utilize a commercially-available device combined with immortalized human RPTEC/TERT1 line—a stable cell line that maintains endogenous expression and functionality of most transporters and metabolic enzymes and has been used extensively for toxicological investigations^2,17,20,24^. The RPTEC/TERT1 immortalized human renal tubule cell line, while applicable to the studies presented here, we acknowledge its limitations. As with any immortalized in vitro cells, the cell line's precision representing the specific cell origin is debatable. Even though the RPTEC/ TERT1 cells were isolated from a healthy male donor and their renal properties were well characterized and functionally similar to proximal tubule cells in the body, it is essential to note that the kidney nephrons are comprised of many different cell types which work together to perform renal functions. Thus, it would be desirable to further define the RPTEC/TERT1 cell line transcriptomic and proteomic profiling compared to other primary renal proximal tubule cells. Our goal was to describe a novel 3D model using transcriptomic and functional assays and demonstrate that this model reproducibly mimics key features of renal proximal tubule-like cells and would thus be a useful tool in future studies of proximal tubule and renal biology.

We first sought to determine whether our proximal tubule cell line showed altered transcriptional profiles under fluid shear stress compared to the same cells grown under static conditions. Our RNA-sequencing data confirmed previously reported shear stress-induced changes in gene expression of CDH1, COX2 (PTGS2), CCL2 (MCP1), EDN1, EGR1, and SNAI1 in renal epithelial cell^14,21–24^. Furthermore, the expression data reveal over 10,400 other genes altered by fluid shear stress in RPTEC/TERT1 cells. Many of these genes have been linked by GWAS (genome-wide association study) to kidney related traits supporting our hypothesis that FSS induces a gene expression pattern that are relevant for normal PTCs function and also relevant for kidney disease^25,26^. In addition, gene set enrichment analysis on the human PTCs showed strong enrichment for anticipated key processes known to be important for PTCs function including cytoskeletal proteins, endocytic functions, and transporters. Some of the up-regulated molecular functions include voltage-gated chloride channel activity and chloride ion binding, as well as, the aldo–keto reductase superfamily. Both chloride channels and their activity are involved in filtration processes in the proximal tubules together with the process of endocytosis. The aldo–keto reductase superfamily, which reduces aldehydes and ketones, have been previously studied in the podocyte cells of the kidney^25–27^. However, with AKR superfamily involvement in proteinuria and other forms of chronic kidney disease, its being recognized as important to the proximal tubules and more research towards a better understanding of the molecular mechanisms behind kidney function^27^. Interestingly enrichment for down-regulated pathways included the cytoskeletal protein, actin, cell adhesion binding, and microfilament motor activity (Fig. 3). These pathways could contribute to the cytoskeletal reorganization of F-actin and endocytic processes involved in albumin uptake. These pathways and other down-regulated genes involved in PTCs function support further examination of cell responses to fluid shear stress. Of note is that several genes induced by shear stress are members of the solute carrier (SLC) family and ABC transporters, which are important for the kidneys role in homeostasis. They have also been hypothesized to play an important role in sensing metabolites secreted by microorganisms, a function central to the remote sensing and signaling hypothesis^28^. In our study, numerous biological pathways, transporters, and drug-metabolizing enzymes were directly impacted by fluid shear stress—many of which are also involved in drug absorption, distribution, metabolism, and elimination.

The in vivo FSS varies from 0.3 to 1.0 dyn/cm^2^ or greater in the proximal tubules of healthy individuals (depending on the segment). Changes in flow rate is common in kidney diseases due to hyperfiltration, tubular dilation, and obstruction, which occurs in functional nephrons, to compensate for lost glomeruli and tubules, with diabetic nephropathy and Polycystic Kidney Disease (PKD) as the most common examples^29^. While some genes are induced with any level of FSS (e.g. AQP4, SLC members and CLDN gene family), other genes show increasing expression with increasing FSS. These may be of particular importance for diseases where FSS is reduced which might impact expression of these FSS-dependent genes like RBP4, ABCG2, CYP1A1, and SLC47A1. Loss of retinol-binding protein 4 in urine (RBP4) is a biomarker for loss of function of the human proximal renal tubule^30^. The efflux transporters, Breast Cancer Resistance Protein (BCRP/ABCG2) and Multidrug and Toxin Extrusion (MATE)-type transporter 1 (MATE1, SLC47A1) are responsible for restricting absorption and enhancing excretion of many pharmaceutical compounds including multiple anticancer drugs^4,31^. Some genes were turned on only by the low- (0.1 dyn/cm^2^) and mid- (0.25 dyn/cm^2^) of flow (FOXA3 and HEY1). Additional exploration of how these genes might be relevant for kidney development or disease is necessary to fully understand this result.

Functional characterization of RPTEC/TERT1 cells grown in our model system under FSS complements our transcriptomic analysis to demonstrate how this model mimics in vivo PTC function. We showed an FSS-dependent increase in formation of tight junctions, an increased albumin uptake, and increased efflux. In our device, tight cell–cell junction proteins were observed between neighboring cells and linked cells in a characteristic cobblestone pattern found *in vivo*^28,29^. Intact actin cytoskeleton (F-actin) is important for regulation of flow dependent ion absorption and endocytosis process of glomerular filtrate proteins is dependent on the integrity of actin cytoskeleton^28,30^. Disruption of F-actin and cytoskeletal organization of PTCs is important to endocytosis the formation of clathrin-coated structures^31^. Additionally, we observed primary cilia in our model, a key feature of PTCs necessary for mechanosensing and regulation of tubular morphology^1,31,32^.

In the human kidneys, PTCs reabsorb filtered solutes and proteins, such as glucose, phosphate, amino acids, and urea, from the glomerular filtrate by secondary active transport; however, they reabsorb proteins such as albumin by receptor-mediated endocytosis^33^. We showed that uptake of cellular FITC-albumin fluorescence was flow-dependent. This is physiologically relevant for future use of the model system because PTCs are responsible for the majority of the glomerular filtrate, and thus, increased expression of these cross epithelial transporters is crucial for its function^11^.

Next to active functional albumin reabsorption and uptake, apical efflux transporters are important to understanding kidney physiology and pharmacological studies^17,21^. Interaction of drugs with the ABC efflux transporters can increase the toxicity of co-administered agents and, in fact, new draft United States Food and Drug Administration guidelines require determination of whether a drug candidate is a substrate or inhibitor of P-gp^34^. We showed ABC mediated transport also showed dependence on FSS. This indicates that kidney tubular epithelial cells under fluidic flow display more effective P-gp and MRP2/4 efflux activity under FSS conditions. Thus, a human PTCs microfluidic device might provide an advantageous in vitro model for renal physiology, kidney diseases research, pharmaceutical and nephrotoxicities studies. While previous studies have demonstrated a change in proximal tubule cell morphology when exposed to fluidic shear stress, this is the first report of a direct comparison in transport activity between static and fluidic culture conditions in a model using the stably immortalized human cells, RPTEC/TERT1.

Overall, this novel in vitro model is valuable for studying renal pharmacology, renal drug transport, and toxicities relevant to the human kidney biology. This device enables direct visualization and quantitative analysis of diverse biological processes similar to the intact kidney tubule in ways that have not been possible in traditional cell culture or animal models.

## Conclusions

This study demonstrates the utility of a 3D model for mimicking in vivo function of human renal proximal tubule cells. We used genomics and functional analysis to show that cells under fluidic shear stress in a 3D fluidics device are capable of critical PTCs functions and display expected PTCs transcriptomic profiles including formation of tight junctions, drug efflux, ion and solute transport, and endocytosis. This is the first transcriptomic analysis of response to FSS in RPTEC/TERT1 cells and the varying flow rate also demonstrates differences that may be relevant for kidney disease. Our in vitro study demonstrates a comprehensive overview of fluid shear stress altered gene expression in human renal epithelial cells, but is not fully representative for the in vivo situation, because of the limitations of immortalized cell lines. Nevertheless, our results and ongoing research of kidney function and disease can benefit from a more biologically relevant PTCs model. This novel in vitro model provides a useful approach for studying renal pharmacology, renal drug transport, and toxicities relevant to the human kidney biology.

## Methods

### Maintenance of cell culture

Human immortalized hRPTECs (RPTEC/TERT1, ATCC CRL-4031) and were cultured and maintained in hTERT Immortalized RPTEC Growth Kit (ATCC ACS-4007), supplemented with Geneticin (Gibco, 10131035), in phenol red free DMEM/F-12 medium (Gibco, 11039021) according to the vendor's instructions. RPTEC/TERT1 cells were passaged 1–2 times per week and subcultured at a 1:2 or 1:3 ratio. Cells were cultured at 37 °C in a humidified atmosphere containing 5% CO2.

### Mimicking the human proximal tubule environment on-a-chip (device setup)

The hRPTECs were grown to confluency in a microfluidic device obtained from the SynVivo Inc. (www.synvivobio.com, Huntsville, AL) using serum free media. The dimensions utilized were liner channel devices with a width of 500 μm, a constant depth of 100 μm, and lengths of 1 cm. Cells are maintained in their growth medium before experiments in the microfluidic device. The device was pre-coated with the extracellular matrix protein, collagen I (A1048301, ThermoFisher) per vendor’s instructions at the concentration of 50 µL/mL. Cells are seeded into the device and are allowed to incubate for approximately 24 h to allow the cells to attach. After this incubation, a syringe pump (PHD ULTRATH, Harvard Apparatus) is programmed to replace the media volume in the device completely with fresh media every 48 h (as under static maintenance conditions). Once the cells reached a confluent monolayer in 72–96 h, channels were placed under multiple levels of fluid shear stress (FSS) conditions, including low flow rates of 0.1dyn/cm^2^ or 0.25 dyn/cm^2^, and a physiological level of 0.5 dyn/cm^2 1^. Controls remained under static conditions. Each biological replicate represents cells within an individual channel in a device. Functional characterization and assays were performed after 24 h of FSS treatment.

### Immunofluorescence

After physiological relevant level (0.5 dyn/cm^2^) FSS stimulation for 24 h, the static and flow channels were quickly rinsed with HBSS, and fixed with 4% paraformaldehyde for 15 min. The fixed cells were permeabilized in 0.1% Triton-X100 and blocked in 5% BSA before being incubated overnight with the antibodies directed against ZO-1 (visualizing tight junction proteins) (Thermo 339100) and acetylated alpha-tubulin (enabling imagining of primary cilia) (Thermo 32-2700).

### Chemicals

Albumin–fluorescein isothiocyanate conjugate (A9771), Valspodar (PSC833) (SML0572), and MK-571 sodium salt hydrate (M7571) from Sigma-Aldrich, US. Calcein-AM and Cell Tracker Green CMFDA (Life Technologies, U.S.A.).

### Functional albumin uptake study

The human RPTEC/TERT1 cells were under physiological relevant level (0.5 dyn/cm^2^) FSS stimulation for 24 h and were removed from the pump immediately before the endocytosis assay. To investigate albumin endocytosis uptake of cells under static and flow conditions, cells were exposed to 50 ug mL^−1^ Fluorescein (FITC)-conjugated albumin (A9771, Sigma) for 15 min at 37 °C in a humidified atmosphere containing 5% CO2. Rinsing with ice-cold HBSS arrested albumin uptake and fluorescence images were obtained using a microscope (Nikon TE-2000). We acquired four fluorescence images of the FITC channel FITC (200 ms exposure) immediately after washes using Photometrics CoolSnap HQ2 Monochrome CCD Camera (Tucson, AZ) with a 20 × /0.75 Plan Fluor Phase Contrast objective, having a total field of 6 × 8. Fluorescence images were analyzed using ImageJ software (NIH, Version 1.51a) to obtain mean fluorescence intensity (MFI) from one FSS channel and one static control channel in four independent devices for each experimental design.

### Efflux transporter assays

The transporter substrates, Calcein-AM (1 μM) and CMFDA (1 μM) (both 2% DMSO vehicle), were incubated with serum free culture media at 37 °C for 60 min or 30 min, respectively after 24 h treatment of FSS. Transport incubation was stopped by placing the samples on ice and washing three times with cold serum free culture media. After washing, the samples were immediately imaged.

To establish fluorescent substrate uptake in the 3D model system, RPTEC/TERT1 cells remained under physiological relevant level (0.5 dyn/cm^2^) FSS stimulation or static conditions for 24 h and were removed from the pump immediately before efflux transporter assays were performed. The transporter substrates, Calcein-AM (1 μM) and CMFDA (1 μM) (both 2% DMSO vehicle), were incubated using serum free culture media at 37 °C for 60 min or 30 min respectively. Transport incubation was stopped by placing the samples on ice and washing three times with cold serum free culture media. Afterward, the samples were immediately imaged. Fluorescent images were obtained as before. Fluorescence images were analyzed using ImageJ software (NIH, Version 1.51a) to obtain mean fluorescence intensity (MFI) from one FSS condition and one static control channel in four independent devices for each experimental design.

### Statistical data analysis

For statistical analyses, Unpaired *t* test was performed using GraphPad InStat software (GraphPad Software Inc., San Diego, CA, USA). All data are presented as means + /- standard error; differences between groups were considered statistically significant when *p* < 0.05.

### RNA-seq sample preparation

RPTEC/TERT1 cells were grown to confluence and exposed to FSS at the different rates as described above. Total RNA was obtained from cells grown in four independent device channels after exposure to flow for 24 h using the ReliaPrep RNA Miniprep Systems (Z6011, Promega) following the manufacturer's instruction.

Total RNA samples were evaluated for concentration by Qubit and for integrity by Bioanalyzer prior to pooling a total of 24 samples over 7 lanes. The RNA-sequencing libraries were generated by the HudsonAlpha Genomic Service Lab (https://gsl.hudsonalpha.org/information/rna) using poly(A) selection and sequencing was performed on an Illumina HiSeq 2500 using paired end reads of 50 bases (Illumina, San Diego, CA, USA) and sequenced an average of 7.6 million reads per sample with an average Q30 score of 94.32%. All samples had an RNA integrity number ranging between 8.8 and 9.8 and 3′ or 5′ bias above 80% by quality control metrics (cite, https://broadinstitute.github.io/picard/). Any samples which did not pass these quality control mapping were not included into the data set^35^.

### RNA-seq analysis

Sequencing reads of 52,463 total genes were aligned using a previously described aRNApipe pipeline (v1.1)^36^. Reads were trimmed with TrimGalore (http://www.bioinformatics.babraham.ac.uk/projects/trim_galore/) prior to alignment with STAR (v2.5.2b)^37^ using the hg37 reference genome. Quality control metrics of the alignment process was assessed with Picard (https://broadinstitute.github.io/picard/). All data analysis in R was performed with R version 3.3.1 using RStudio (v1.1.453). To examine gene expression changes, differential expression was determined by using DESeq2 package (v1.12.4)^38^ using the default settings in likelihood ratio test (LRT) mode^39^. The most highly differentially expressed gene subset list highlighted in the volcano plot (p-adjust ≥ 1 × 10^–30^ and log2FC >  ± 1) was filtered by genes found to be associated with both PTs and kidney GWAS. To generate gene list containing genes that were previously published in association with the search term proximal tubule, the search engine Geneshot was utilized^40^. In order to compile and filter GWAS that identified specific gene to human kidney disease resistance/susceptibility, the complete 12/16/2019 release of the NHGRI-EBI GWAS database was downloaded from the NHGRI-EBI Catalog website (https://www.ebi.ac.uk/gwas/docs/file-downloads). The *H. sapiens* pathway analysis was conducted using functional enrichment analysis web tool, LRPath with enrichment method GSEA and enrichment category gerontology biological process (no redundant) and advance parameters minimum number of genes equal 20^41–43^. Pathway analysis was run using LRPath^41,44^ and pathway analysis was carried out by the top 50 GO terms by p-value separating up- and down-regulated GO terms and visualized using REVIGO (Reduce and Visualize Gene Ontology) available online and run using default parameters^19^. Gene IDs were converted to ENSEMBL gene IDs using R packages biomaRt (v.2.28.0) and biomartr (v.0.7.0)^45^. The R packages pheatmap (v.1.0.10), ggplot2 (v. 3.0.1), edgeR (v.3.14.0), and ggfortify (v.0.4.4) were used for figure preparation.

## Supplementary Information

Supplementary Information 1.

Supplementary Information 2.

Supplementary Information 3.

Supplementary Information 4.

Supplementary Information 5.

Supplementary Information 6.

Supplementary Information 7.

Supplementary Information 8.

## Data Availability

The RNA sequencing data discussed in this publication have been deposited in NCBI's Gene Expression Omnibus (Edgar et al., 2002) and are accessible through GEO Series accession number GSE172062 (https://www.ncbi.nlm.nih.gov/geo/query/acc.cgi?acc=GSE172062).
