# Supplementary material for: Three dimensional modeling of biologically relevant fluid shear stress in human renal tubule cells mimics in vivo transcriptional profiles
Source: Sci Rep. 2021 Jul 7;11:14053. doi: 10.1038/s41598-021-93570-5 (PMC8263711; doi:10.1038/s41598-021-93570-5)
Supplement: Supplementary file 1 — Supplementary Information 1. [file 41598_2021_93570_MOESM1_ESM.pdf]

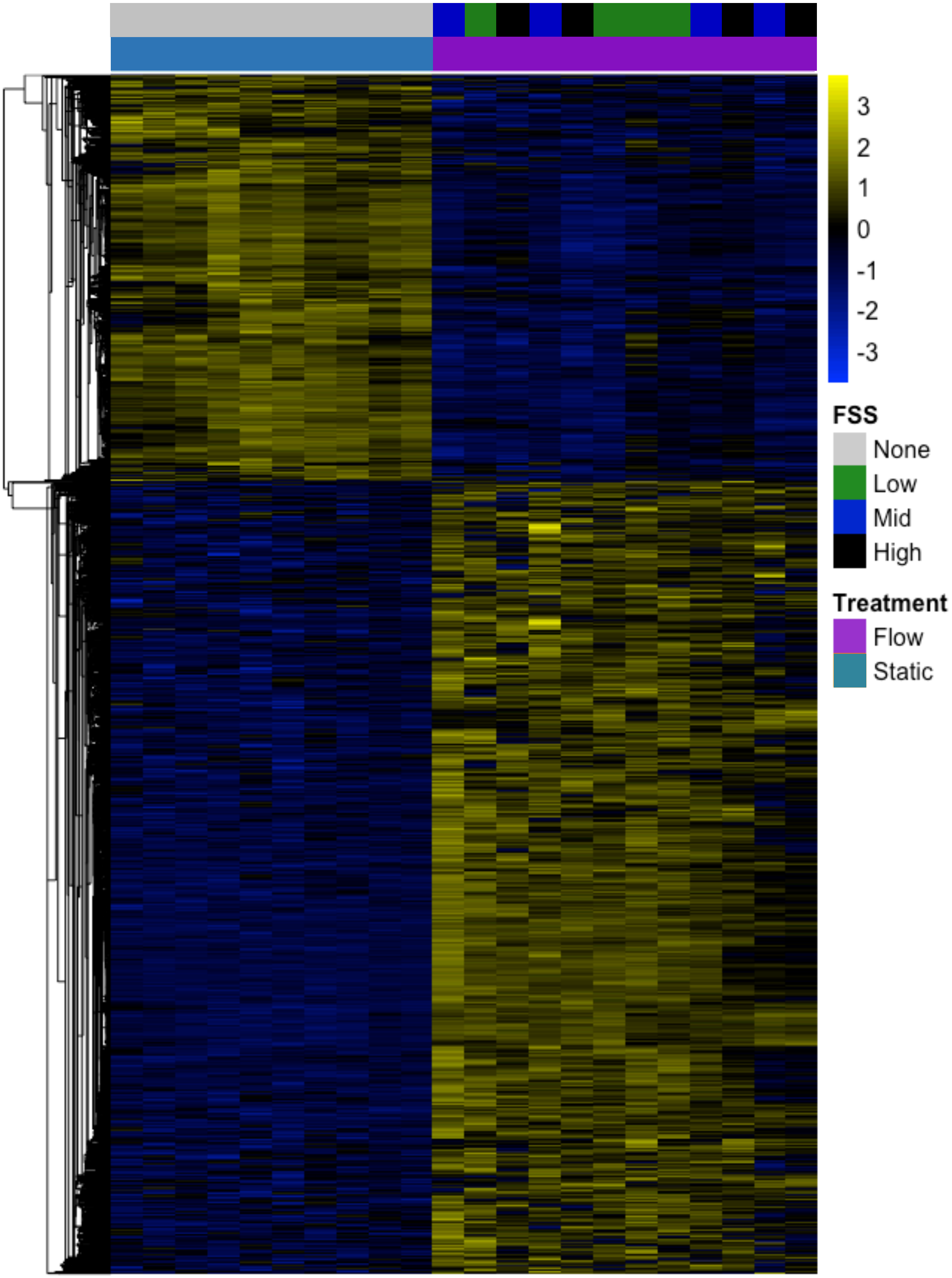

Supplemental Figure 1: Heatmap representing gene expression level. Gene expression profiling shows a strong difference between fluid shear stress treatment RPTEC/TERT1 (purple) and static controls (teal). Heat map showing the expression values (log2FC±1; padj < 0.05) in increasing flow rates 0.1 dyn/cm^2 (green), 0.25 dyn/cm^2 (blue), and 0.5 dyn/cm^2 (biological, black) fluid shear stress treated samples (FSS) and corresponding static controls (none, gray). Expression values were normalized using DESeq2 R package.
